# Fine Particulate Matter (PM_2.5_) Sources and Its Individual Contribution Estimation Using a Positive Matrix Factorization Model

**DOI:** 10.3390/toxics11010069

**Published:** 2023-01-11

**Authors:** Gahye Lee, Minkyeong KIM, Duckshin Park, Changkyoo Yoo

**Affiliations:** 1 Seohaean Research Institute, Chung Nam Institute, Hongseong 32227, Republic of Korea; 2 Korea Railroad Research Institute (KRRI), 176 Cheoldobakmulkwan-ro, Uiwang-si 16105, Republic of Korea; 3 Department of Environmental Science and Engineering, Kyung Hee University, Yongin 17104, Republic of Korea

**Keywords:** conditional probability function, PM_2.5_, positive matrix factorization, receptor method

## Abstract

The effective management and regulation of fine particulate matter (PM_2.5_) is essential in the Republic of Korea, where PM_2.5_ concentrations are very high. To do this, however, it is necessary to identify sources of PM_2.5_ pollution and determine the contribution of each source using an acceptance model that includes variability in the chemical composition and physicochemical properties of PM_2.5_, which change according to its spatiotemporal characteristics. In this study, PM_2.5_ was measured using PMS-104 instruments at two monitoring stations in Bucheon City, Gyeonggi Province, from 22 April to 3 July 2020; the PM_2.5_ chemical composition was also analyzed. Sources of PM_2.5_ pollution were then identified and the quantitative contribution of each source to the pollutant mix was estimated using a positive matrix factorization (PMF) model. From the PMF analysis, secondary aerosols, coal-fired boilers, metal-processing facilities, motor vehicle exhaust, oil combustion residues, and soil-derived pollutants had average contribution rates of 5.73 μg/m^3^, 3.11 μg/m^3^, 2.14 μg/m^3^, 1.94 μg/m^3^, 1.87 μg/m^3^, and 1.47 μg/m^3^, respectively. The coefficient of determination (R^2^) was 0.87, indicating the reliability of the PMF model. Conditional probability function plots showed that most of the air pollutants came from areas where PM_2.5_-emitting facilities are concentrated and highways are present. Pollution sources with high contribution rates should be actively regulated and their management prioritized. Additionally, because automobiles are the leading source of artificially-derived PM_2.5_, their effective control and management is necessary.

## 1. Introduction

Among member countries of the Organization for Economic Cooperation and Development, the concentration of fine particulate matter—an atmospheric pollutant—in the Republic of Korea was recently reported to be the highest [1]. Although the Republic of Korea met its national standard for concentrations of fine particulate matter less than 2.5 μm in diameter (PM_2.5_; 15 μg/m^3^ or less) in 2020, it failed to meet the recommended World Health Organization target of 10 μg/m^3^ [2].

Human industrial and/or mechanical activities in rapidly-developing societies produce fine particulate matter [3], with such particles increasing the risks of disease and death as well as posing other threats to human health. Microparticles carrying heavy metals, sulfates, and nitrates are particularly influential in causing or exacerbating cardiovascular and respiratory diseases, cancer, and mental illness, resulting in serious adverse effects on the human body [4]. Moreover, ultrafine particulate matter directly penetrates cells, leading to severe damage [5]. Accordingly, long-term measures and policies to reduce PM_2.5_ concentrations are needed in the Republic of Korea, as these particles are more harmful to the human body than coarser particles such as PM_10_.

The Republic of Korea has introduced many management policies to reduce PM_2.5_ and uses distributed models and other methods to predict future air quality. Nevertheless, it is essential to identify major pollution sources and assess their contributions with an acceptance model to effectively predict and manage PM_2.5_ concentrations, given that the chemical composition and physicochemical properties of PM_2.5_ vary according to its spatiotemporal characteristics [6]. However, in the Republic of Korea, research is mainly limited to large areas such as Seoul and Jeju Island, and the majority of observational studies have focused on specific events [7].

“Air control zones” refer to areas with severe air pollution due to emissions from the capital region. The Republic of Korea has recently categorized the central, southeastern, and southern regions of the country as air control zones, with these zones including a total of 77 special and metropolitan cities and municipalities. For this study, Bucheon City in Gyeonggi Province, an air control zone, was selected as a PM_2.5_ data collection area. The chemical composition of the particulate matter in this area was analyzed and its physical and chemical properties identified. The positive matrix factorization (PMF) model, the most commonly used receptor model worldwide, was employed to analyze the chemical contents. The PMF model does not rely on a correlation matrix of measured data but, rather, a weighted least squares fitting algorithm based on estimates of errors in the measured data and measurement information. It can accommodate data representing values below the detection limit (BDL) and estimate missing data through error evaluation. The model also minimizes the number of discarded measurements. The aims of the study were to identify the main sources of PM_2.5_ and quantitatively estimate the contribution of each pollution source through PMF modeling. Conditional probability function (CPF) plots were generated to identify the potential locations of pollutants arriving from various wind directions using the pollutant contribution data calculated with the PMF model and meteorological data. The results of this study can help with more effective management of PM_2.5_ generated not only in this study area, but also in other urban areas in the Republic of Korea, even without a pollutant classification table. Moreover, the data generated can be used as reference data for establishing measures to reduce fine particulate matter and improve air quality.

## 2. Materials and Methods

### 2.1. Research Period and Sampling Points

PM_2.5_ samples were collected in Bucheon City, Gyeonggi Province, from 22 April to 3 July 2020. Bucheon City is a satellite city located adjacent to the southwestern part of Seoul Metropolitan City, east of Incheon Metropolitan City, and north of Siheung City. It is situated in the middle of the Korean Peninsula along the north–south axis and has a total area of 53.4 km^2^, accounting for 0.5% of Gyeonggi Province’s total area. According to Article 13 of the Enforcement Decree of the Republic of Korea Air Environment Conservation Act, business sites are classified into one to five types according to the number of pollutants generated from discharge facilities. Class 1 business refers to businesses with an annual total of more than 80 tons of air pollutants; Class 2 businesses are those with an annual amount of more than 20 tons to less than 80 tons; Class 3 businesses are those with an annual amount of more than 10 tons; and Class 4 businesses are those with an annual amount of more than 2 tons to less than 2 tons. Therefore, there are a total of 755 business sites emitting environmental air pollutants as of July 2020: 3 class 3 sites, 195 class 4 sites, and 558 class 5 sites in this research area. Table 1 shows the status of pollutant-emitting sites in each administrative district in Bucheon City.

**Figure 1:**
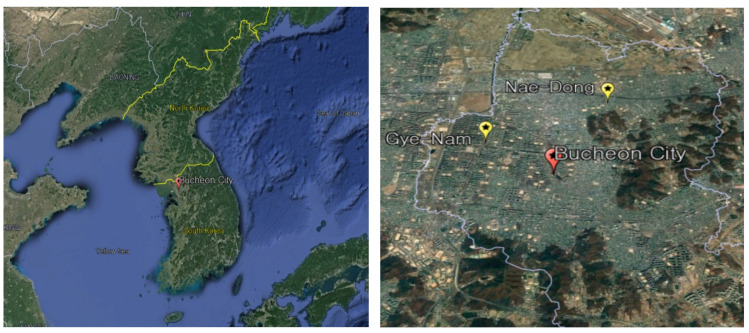
Locations of the study area and sampling points.

Two sampling points were selected: the Songnae-daero monitoring station (Gyenam Park) in Jung-dong and the Nae-dong monitoring station in Samjeong-dong, located approximately 2.5 km apart (Figure 1).

### 2.2. Sampling and Analysis

Samples were collected using four sequential particulate matter samplers (Low-Volume Air Sampler, model PMS-104, APM Engineering Co., Ltd., Bucheon-si, Republic of Korea); at each sampling point, one sampler was placed within the monitoring station and one was placed outside. Each PMS-104 instrument sampled at a rate of 16.7 L/min over 24 h. A total of 74 samples—40 from Gyenam Park and 34 from Nae-dong—were collected. Each sampler was equipped with a Teflon filter (2.5-μm PTFE membrane, 46.2 mm, Tisch Scientific, Cleves, OH, USA) pretreated for ion and heavy metal content analysis and a quartz filter (QM-A, 47 mm, Whatman PLC, Little Chalfont, UK) heat treated for carbon content analysis. Each Teflon filter was stored in an electronic desiccator (model 08-642-23C, Fisher Scientific, Waltham, MA, USA) under constant temperature (25 ± 3 °C) and humidity (35 ± 5%) for 72 h to remove moisture before and after sampling. After drying to a constant weight with static electricity removed, the filters were weighed using an electronic balance (model XP6, No. 1123430327, Mettler Toledo, Seoul, Republic of Korea) with a sensitivity of 0.001 mg. Each quartz filter was heat treated in a furnace at 600 °C for about 6 h to remove organic matter that might have been present in trace amounts before sample collection. After the complete removal of organic matter, each filter was placed on a Petri dish, sealed with parafilm, and stored in a desiccator before sampling. After sample collection, the filters were stored in a freezer (<−20 °C) to minimize the volatilization of particulate matter and effects from external factors. Analysis took place immediately once a certain number of samples were collected.

To analyze the water-soluble ionic content of PM_2.5_, the filter on which dust was collected was first placed into a 50-mL beaker [9]. Then, 30 mL of tertiary distilled water was added to precipitate the particulate matter. Next, the sample was transferred to an ultrasonic extractor (model WUC-D03H, Daihan Scientific Co., Wonju-Shi, Republic of Korea) and eluted after mechanical shaking and ultrasonication extraction for 30 min. The extracted solution was filtered through a sterile membrane filter (Whatman PLC, UK) with a diameter of 47 mm (pore size 0.45 μm) to remove insoluble particles. Finally, to prepare for ion analysis, the solution was transferred to a vial and only the ionic component was eluted. Ion chromatography (model 861, Advanced Compact IC, Metrohm AG, Herisau, Switzerland) was used to analyze the water-soluble ion contents of the following 11 components in PM_2.5_: Br^−^, PO_4_^3−^, F^−^, Cl^−^, NO_3_^−^, SO_4_^2−^, Na^+^, NH_4_^+^, K^+^, Ca^2+^, and Mg^2+^.

X-ray fluorescence (XRF) spectrometry (S2 Ranger X-ray spectrometer, Bruker, Germany) was used to analyze inorganic elemental contents in PM_2.5_. Because XRF spectrometry is a non-destructive analytical method that does not damage filters, no pretreatment was necessary. The following 22 elements were analyzed: Al, Ti, Co, V, Se, Sn, As, SiO, Cl, Mg, Zn, K, S, Br, Ca, Ba, Pb, Cr, Cu, Mn, Fe, and Ni.

For carbon analysis, the thermal–optical transmittance method conforming to the National Institute of Occupational Safety and Health 5040 standard and following a temperature-specific protocol was employed. A thermos–optical OCEC analyzer (Sunset Laboratory Inc., Portland, OR, USA) was used to analyze organic carbon (OC) and elemental carbon (EC).

### 2.3. PMF Model

The PMF model is used in the field of applied statistical analysis. It considers standard deviation and measurement uncertainty through a mathematical algorithm that enhances negative factor loading during factor analysis. Hence, factor loads are calculated only as positive values [10], making it possible to quantitatively estimate pollutant emissions [11]. The basic PMF model is as follows:

(1) X=GF+E

where *X* can be expressed as an m × n matrix, with m representing the chemical species analyzed among n samples. Therefore, matrix *X* is structured as rows comprising the concentrations of chemical species across samples and columns that each represent a collected sample. *G* is an n × *p* matrix representing the contribution of a pollutant source to each sample. *F* is a *p* × m matrix representing the source profile for a specific pollutant. Here, *p* refers to the number of extracted elements. Each column in *G* then refers to the amounts of a specific pollutant emitted. *E* is a residual matrix and is expressed as shown in Equation (2) below. The most important step in PMF is to determine the appropriate number of elements, and a useful method is to minimize the *Q* value by repeatedly assigning a weight factor to *G* and *F* elements. The *Q* value can be obtained as follows:

(2) $$E_{ij\;}=X_{ij}-\overset{p}{\underset{h=1}{\sum }}G_{ih}F_{hj}\;\left(i=1\sim n,\;j=1\sim m,\;k=1\sim p\right)$$

(3) $$Q=\overset{n}{\underset{i=1}{\sum }}\overset{m}{\underset{j=1}{\sum }}\left(\frac{E^{2}{}_{ij}}{\sigma^{2}{}_{ij}}\right)=\overset{n}{\underset{i=1}{\sum }}\overset{m}{\underset{j=1}{\sum }}\frac{{\left(x_{ij}-g_{ik}f_{kj}\right)}^{2}}{\sigma^{2}{}_{ij}}$$

EPA PMF version 5.0 (US Environmental Protection Agency) was used to run the PMF analysis. The raw data matrix (74 × 35) was reconfigured as part of data pretreatment. A sample with a difference of more than ± 50% between the PM_2.5_ mass concentration and the sum of all chemical contents was removed. Additionally, three species associated with low reliability—Sn, Cu, and F^−^—and two chemical species—Ba and PO_4_^3−^—which had BDL values or missing data in 90% or more of all acquired samples, were not included in the analysis. For species that were analyzed using both ion chromatography and XRF spectrometry, the data that produced better results in the regression analysis were selected for PMF.

Data on PM_2.5_ mass concentrations, the analyzed contents in each sample, and the uncertainty associated with each analyzed concentration are required to perform PMF modeling. Missing concentration data were replaced with geometric mean values, and the corresponding error matrix values were replaced with values of four times the geometric mean. If an analyzed concentration was 0 or BLD, it was replaced by the method detection limit (MDL)/2, with the corresponding error matrix value replaced by (5/6) × MDL. In addition, the error matrix corresponding to the calculated concentration values was derived using $\sqrt{{\left(species\;data\times 0.1\right)}^{2}+{\left(\frac{1}{2}\times MDL\right)}^{2}}$, whereas the error matrix corresponding to PM_2.5_ mass concentrations was calculated by multiplying the mass concentration values by four. The original raw data matrix was reconfigured over several steps into a 73 × 25 matrix. Table 2 shows the final input data used for PMF modeling.

Determination of the optimum number of factors is a critical step in the process of PMF modeling. Since the PMF model can be interpreted differently depending on the number of pollutants, repeated modeling should be performed (trial and error) to determine the optimal number of physically meaningful pollutants [12]. The most commonly used methods in determining the optimal number of pollutants in the PMF model include using the scaled residual matrix *R* and using the *Q* value. Since the purpose of the PMF model is to minimize the *Q* value, it is calculated under the condition that the *Q* value is minimized among the results modeled by various input variables. In the method of using the scaled residual matrix, the probability that the standardized residual value is in the interval between −3.0 and +3.0 must be at least 80%. The residual matrix R can be expressed as the following Equation (4).

(4) $$R_{ij}=\frac{E_{ij}}{S_{ij}}$$

In addition, two variables, i.e., a maximum independent column mean (*IM*) value and a maximum independent column standard deviation (*IS*) value, can be calculated from the residual matrix *R* through Equations (5) and (6).

(5) $$\mathrm{IM}=\underset{j=1\ldots m}{\max}(\frac{1}{n}\overset{n}{\underset{i=1}{\sum }}r_{ij})$$

(6) $$\mathrm{IS}=\underset{j=1\ldots m}{\max}(\sqrt{\frac{1}{n}\overset{n}{\underset{i=1}{\sum }}{\left(r_{ij}-\bar{r_{i}}\right)}^{2}}$$

**Figure 2:**
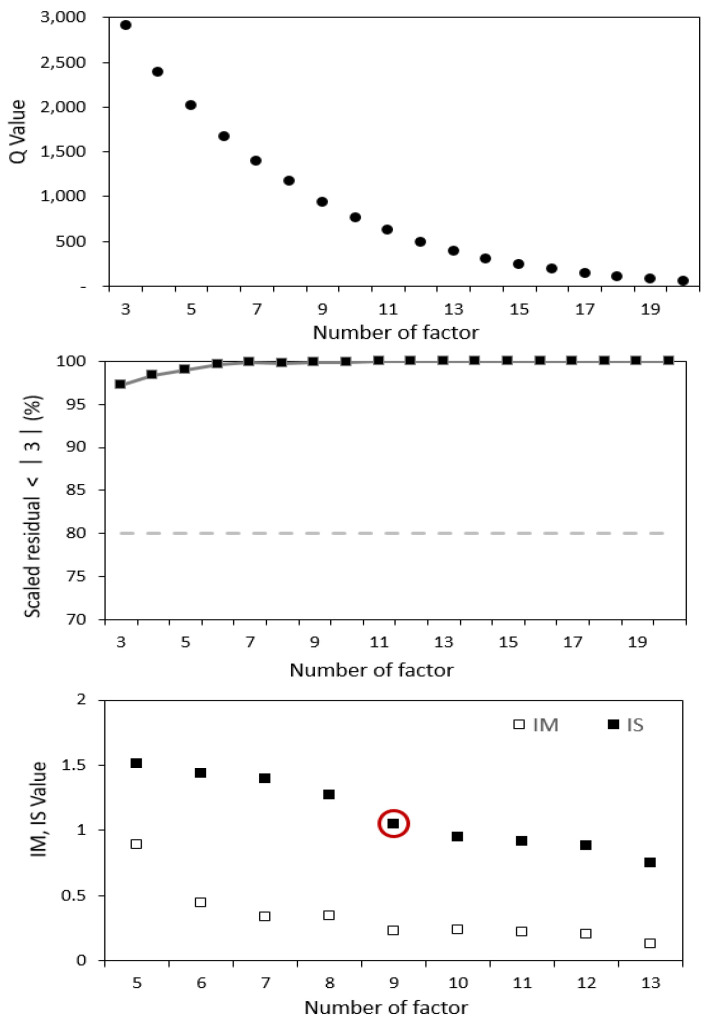
The Q value, scaled residual, IM, and IS for determining the number of factors.

When the number of factors increases to a threshold value, the *Q* value decreases, and the *IM* value and the *IS* value also decrease rapidly (Figure 2). As a result, the optimal number of pollutants was determined to be 9.

Thereafter, changes in the value of *Q* with each rotation of elements were checked every 0.1 steps from −1.0 (instead of 0) to 1.0 to determine the optimal Fpeak value for converting the results of the derived final model into a simple form. The most physically meaningful result was derived when the Fpeak value was −0.1.

### 2.4. CPF Model

The CPF model can be used to estimate the potential locations of emitted pollutants arriving from different wind directions by applying pollutant contribution data, calculated using the PMF model and meteorological data, to evaluate the local impacts of pollutants [13,14,15,16]. In *CPF* analysis, conditional probability values for different wind directions for a reference concentration can be calculated as follows:

(7) $$CPF_{\Delta \theta }=\frac{m_{\Delta \theta }}{n_{\Delta \theta }}$$

where ∆*θ* refers to a particular wind direction sector, *n*∆*θ* refers to the total frequency of wind blowing through ∆*θ*, and *m*∆*θ* refers to the number of times the pollutant concentration exceeds the appropriate standard when the wind is blowing through ∆*θ*.

The 80th percentile concentration value was used as the critical concentration standard and low wind speeds (≤1 m/s) were excluded as, at such speeds, conditions are considered relatively “windless”, during which uncertainty about the wind direction is high. Because meteorological data for the Republic of Korea include 16 compass directions for wind direction, the number of wind direction sectors was determined to be 22.5.

## 3. Results and Discussion

### 3.1. Source Classification and Identification through PMF Modeling

**Figure 3:**
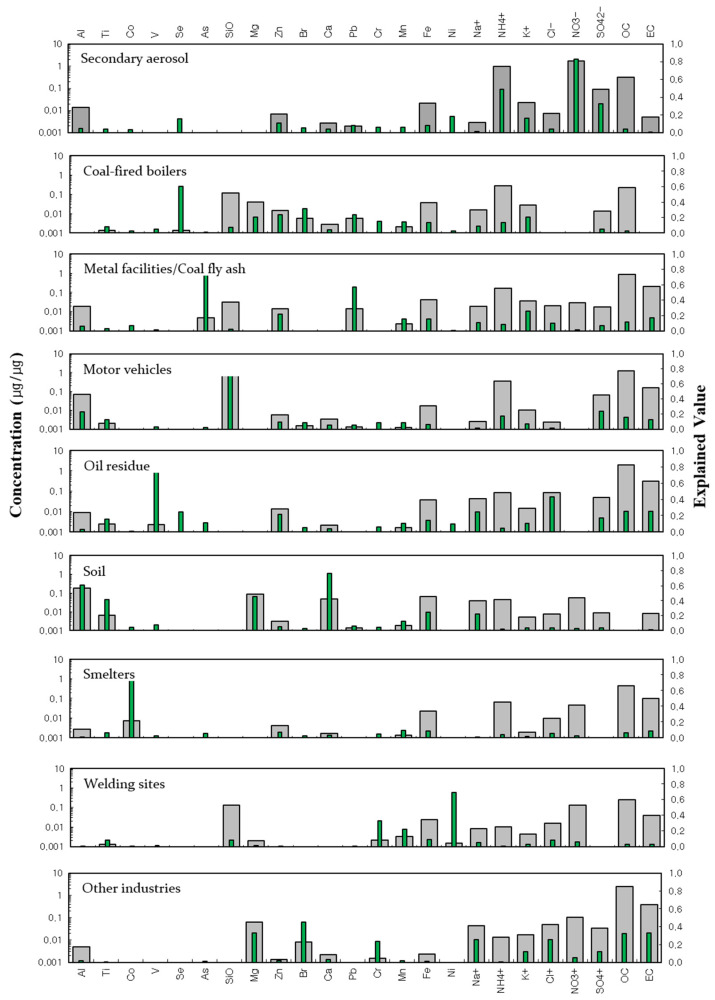
Source profile resolved from PM_2.5_ in the study area. Gray: Concentration of species.Green: Percentage of species (%).

Pollution sources were identified by reviewing source profiles compiled from the analysis of the concentration and proportion (%) of each chemical species and the time series of elemental change patterns. Figure 3 shows the classification of pollutants based on modeling in this study.

Nine pollution sources were identified, of which the largest was that of secondary aerosols, comprised mostly of SO_4_^2−^, NO_3_^−^, and NH_4_^+^. These species are considered secondary pollutants, with SO_4_^2^ produced from homogeneous and non-uniform reactions in air and NO_3_^−^ produced from uniform reactions of gaseous HNO_3_, which is generated from photochemical reactions and bonds strongly with NH_4_^+^ [17,18]. These pollutants travel over long distances and can be derived from the conversion of aqueous solution states, such as in-cloud conversion [19].

The next largest pollution source was estimated to be that of coal-fired boilers, which release emissions containing Se, Br, Pb, Mg, Zn, and K^+^. Although coal consumption has decreased gradually in recent years, coal is still used in many industrial boilers and furnaces [20]. The third-ranked pollution source was that of metal-processing facilities and coal fly ash related to the processing of iron and non-ferrous metals, which release Pb, As, K^+^, and Zn as the main pollutants. Motor vehicles ranked fourth. The typical chemical components in automobile-derived pollutants are EC, OC, SO_4_^2−^, NH_4_^+^, and Si. Accordingly, these were found in the pollutant mix in this study. Around the study area, there is a high density of commercial districts, including a subway station and an intersection between Seoul’s First Ring Expressway and Gyeongin Expressway; thus, traffic in the area is heavy and the floating population is large. This heavy traffic resulted in the classification of motor vehicles as the fourth-ranked pollution source.

The fifth-ranked pollution source was that of oil combustion residues from factories of all sizes in the study area. Pollutants were generated from the combustion of diesel or Bunker-C oil or the incomplete combustion of solid fuels, resulting in V, OC, EC, Na, and Cl being released into the air. Soil, which was found to release heavy metals such as Al, Ca, Mg, Ti, and Fe, was classified as the sixth-ranked pollution source. These elements are typically used to track soil-related pollution and were thus categorized as soil-derived pollutants in this study. Particles distributed in soil can be scattered onto roads, while those on sidewalks can be scattered into the atmosphere, including yellow dust. This study was conducted in spring and summer, when yellow dust is an issue [21].

Smelters—including rotary kilns and blast furnaces—in steelmaking and smelting facilities processing iron ore and non-ferrous metals near the study area were categorized as the seventh-ranked pollution source. Co was the largest contributor, whereas As, Zn, Mn, and Fe were secondary contributors. Together, these elements were the main indicators of pollutants produced by rotary kilns and blasting furnaces. The eighth-ranked pollution source was that of welding sites, with Ni, Mn, and Cr the primary metal components generated at steel-welding facilities. Finally, the ninth-ranked pollution source consisted of other industrial sites, which generated high concentrations of Br, Mg, total carbon, Na^+^, Cl^-^, and Cr. Indeed, many air-pollutant-emitting industrial sites, especially manufacturing plants, are located near the study area.

### 3.2. Quantitative Assessment of the Contribution of Each Pollution Source

**Figure 4:**
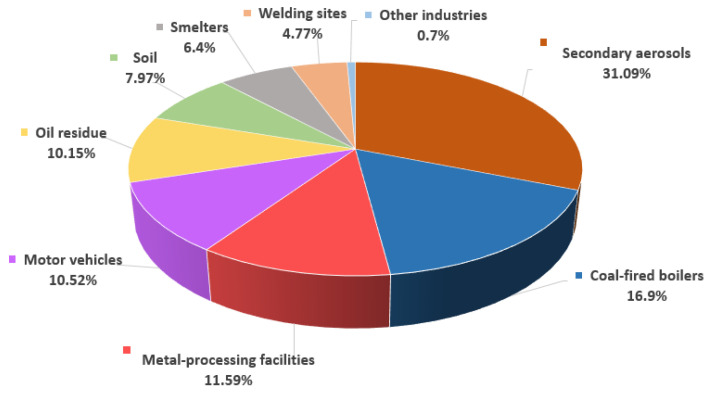
Contribution (%) of each identified source to PM_2.5_ pollution.

The contribution of each pollution source was estimated using a scaled G matrix. Figure 4 shows the average mass contribution rate of each confirmed pollution source to PM_2.5_ collected in the study area from 22 April to 3 July 2020.

Secondary aerosols, coal-fired boilers, metal-processing facilities and coal fly ash, motor vehicle exhaust, oil combustion residues, contaminated soil, smelters, welding sites, and other industries had average contribution rates of 31.09% (5.73 μg/m^3^), 16.9% (3.11 μg/m^3^), 11.59% (2.14 μg/m^3^), 10.52% (1.94 μg/m^3^), 10.15% (1.87 μg/m^3^), 7.97% (1.47 μg/m^3^), 6.4% (1.18 μg/m^3^), 4.77% (0.88 μg/m^3^), and 0.7% (0.11 μg/m^3^), respectively.

As secondary aerosols contributed the most to PM_2.5_ pollution (31.09%, about 1/3 of the total), steps to manage this pollution source must be taken. Additionally, a large proportion of pollutants appeared to be emitted from workplaces. It is likely that many air-pollutant-emitting sites are located near the study area. Standards for air pollutant emissions are urgently needed, and other actions can include restrictions on coal use or the replacement of coal with cleaner fuels. Because motor vehicles are another leading source of pollutants, specific measures such as restrictions on vehicle exhaust emissions, banning the use of old diesel vehicles, and the establishment of no-driving-day schemes for passenger vehicles (e.g., no driving every 2nd, 5th, or 10th day) should be introduced in the study area.

**Figure 5:**
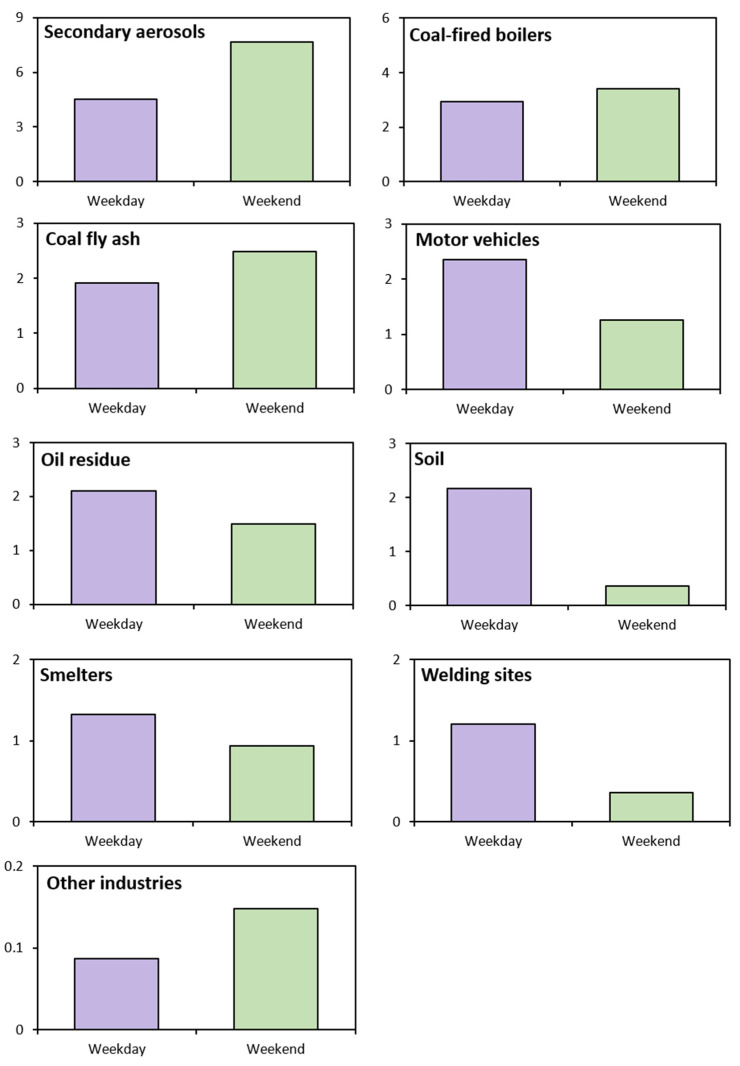
Difference in source contribution to PM_2.5_ between weekdays and weekends.

**Figure 6:**
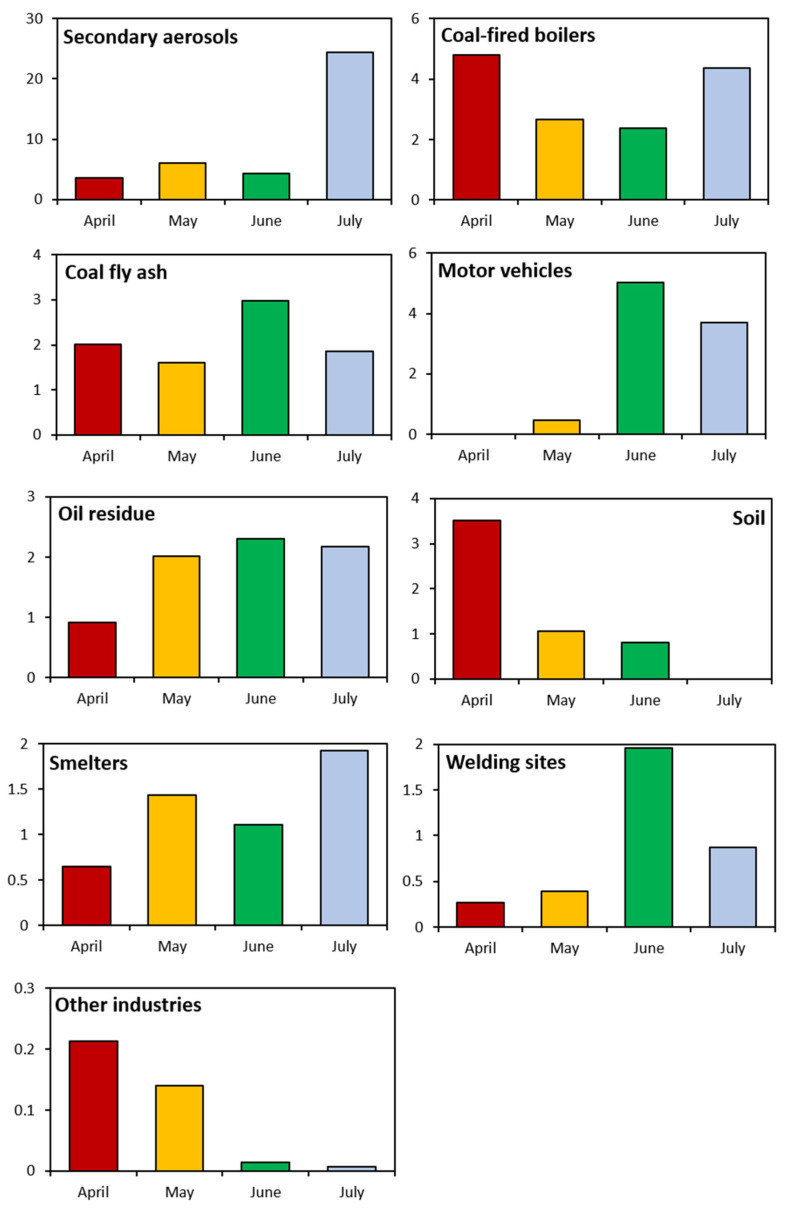
Monthly variation in source contribution to PM_2.5_.

The consideration of the seasonal variation in the analysis of pollutant contributions can ensure greater reliability in identifying pollutant sources. Accordingly, the contribution of each pollution source to the average PM_2.5_ mass was estimated for each month and on weekdays versus weekends. Since the amount of data in July is smaller than in other months, there is a limit to providing accurate information in July. Therefore, it is difficult to generalize that the July contribution data are the average contribution in July, but the contribution was analyzed including July as a reference (Table 3 and Table 4, Figure 5 and Figure 6). In Table 3 and Table 4, the first row of each pollutant refers to the concentration of the pollutant, and the second row refers to the percentage of each pollutant.

In July, May, April, and June, secondary aerosols accounted for 62.17% (24.35 μg/m^3^), 38.14% (6.03 μg/m^3^), 22.66% (3.61 μg/m^3^), and 20.81% (4.36 μg/m^3^) of total pollutants, respectively. The largest contribution was recorded in July, likely because of the active generation of secondary aerosols from photochemical reactions due to intense solar radiation in summer [22]. The percentage contribution was also higher on weekends than on weekdays (Figure 6).

For pollutants derived from coal-fired boilers, the contribution rates in April, May, June, and July were 30.04% (4.79 μg/m^3^), 16.89% (2.67 μg/m^3^), 11.40% (2.39 μg/m^3^), and 11.13% (4.36 μg/m^3^), respectively. The contribution rate was highest in April, when temperatures are lower than in summer. Despite the recent trend of gradually decreasing coal consumption, the high contribution rate in April can be attributed to greater consumption of coal for heating in winter/early spring. Similar to secondary aerosols, the contribution rate was higher on weekends than on weekdays.

For pollutants derived from metal-processing facilities and coal fly ash, the highest contribution rate was observed in June (14.23%, 2.98 μg/m^3^), followed by April (12.56%, 2.00 μg/m^3^), May (10.13%, 1.60 μg/m^3^), and July (4.74%, 1.86 μg/m^3^). There was no significant difference in the contribution rate between weekdays and weekends.

Motor vehicle pollutants contributed to 23.98% (5.02 μg/m^3^), 9.46% (3.70 μg/m^3^), 3.04% (0.48 μg/m^3^), and 0% (0 μg/m^3^) of total pollutants in June, July, May, and April, respectively. The contribution rate on weekdays was higher than on weekends.

Pollutants from oil combustion residues accounted for 12.71% (2.01 μg/m^3^), 11% (2.3 μg/m^3^), 5.72% (0.91 μg/m^3^), and 5.56% (2.18 μg/m^3^) of total pollutants in May, June, April, and July, respectively. Although the contribution rate of these pollutants is generally high in winter due to oil use for heating [23], large manufacturing plants and small factories also use significant amounts of fuel. The contribution rate was higher on weekdays than on weekends, probably because the nearby factories operate primarily on weekdays.

The contribution rates of soil-derived pollutants were 22.05% (3.52 μg/m^3^), 6.64% (1.05 μg/m^3^), 3.89% (0.81 μg/m^3^), and 0% (0.0 μg/m^3^) in April, May, June, and July, respectively. That the contribution rate was highest in April was likely due to yellow dust being present in the study area in April and May. Yellow dust has a significant effect on soil-derived pollution in spring. The contribution rate was higher on weekdays than on weekends, indicating the presence of many industrial facilities near the study area. The higher rate may also be due to particulate matter on sidewalks dispersing into the air via the activities of the large floating population on weekends.

The highest contribution rate of 6.64% (1.05 μg/m^3^) for pollutants from smelters was recorded in May. No significant differences in the rate were observed between April, June, and July. The contribution rate was higher on weekdays than on weekends due to kilns, furnaces, and other industrial facilities operating on weekdays rather than on weekends.

The contribution rate of welding-derived pollutants was highest in June (9.33%, 1.95 μg/m^3^), whereas no significant differences were observed between April, May, and July. The contribution rate was higher on weekdays, when workplaces operate, than on weekends, when they do not operate.

The contribution rates of other industrial pollutants in April, May, June, and July were 1.33% (0.21 μg/m^3^), 0.88% (0.14 μg/m^3^), 0.07% (0.01 μg/m^3^), and 0.02% (0.01 μg/m^3^), respectively. There was no significant difference in the monthly rate between weekdays and weekends, indicating that these pollutants continuously affect the study area. Although many air pollutants contribute to the overall PM_2.5_ concentration, oil combustion residues and motor vehicles were found to be the major pollution sources, implying that intensive management is needed to reduce emissions from these sources.

### 3.3. Assessing PMF Model Reliability

**Figure 7:**
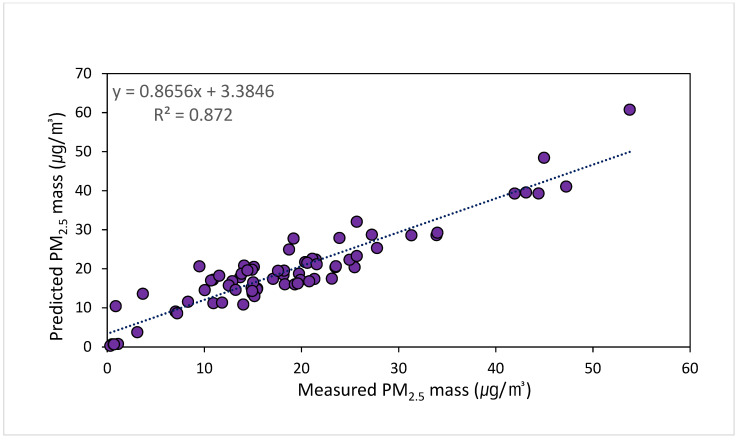
Mass concentrations of PM_2.5_ predicted from PMF modeling versus measured mass concentrations.

It is important to verify the reliability of results of PMF modeling. This can be completed via correlation analysis of the estimated and actual mass concentrations of secondary aerosols [12]. Figure 7 displays a scatter plot of measured PM_2.5_ concentrations and concentrations calculated using PMF modeling during the study period. The coefficient of determination (R^2^) was 0.872, indicating that the estimated PM_2.5_ concentrations can explain 87.2% of the variability in actual concentrations.

### 3.4. Using CPF Modeling to Verify the Source Locations of Pollutants

**Figure 8:**
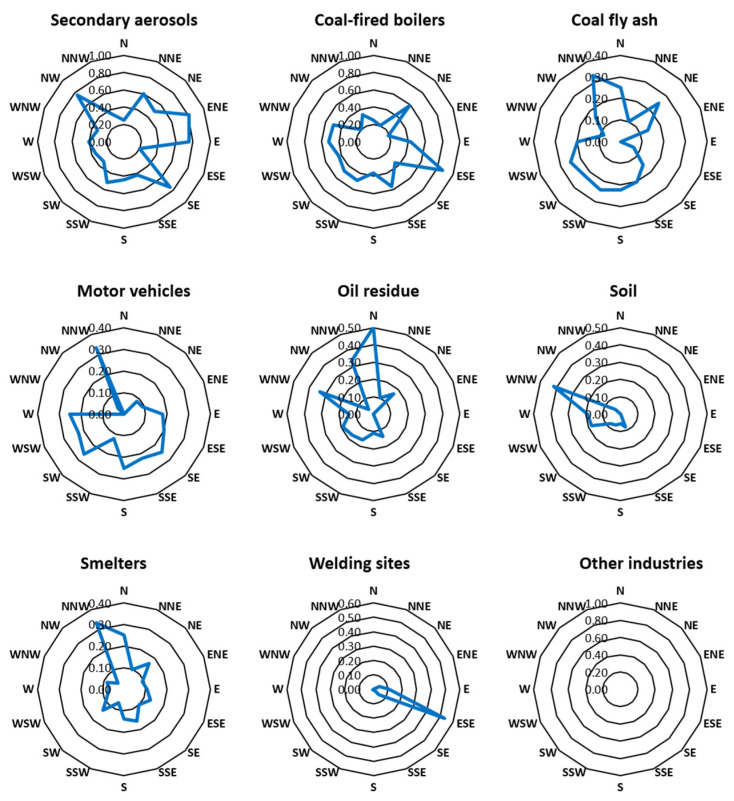
CPF plot for each source of PM_2.5_ identified in the PMF analysis.

CPF modeling was used to evaluate the directions from which incoming identified came by combining the contribution rates calculated from the PMF model with detailed weather observation data (AWS; Automatic Weather Station, which automatically transmits or records observations obtained from the measuring instrument.) by region. Figure 8 shows the results of CPF modeling.

Secondary aerosol pollutants largely came from the east and northwest, likely due to vehicles traveling along highways in the northwest and emissions of SOx from facilities of various sizes in the east. Additionally, because secondary aerosol pollutants are generated through chemical reactions, they may originate from far away. Therefore, it is possible that some of the aerosols were generated in China.

Pollutants from coal-fired boilers were mostly transported from the southeast. Indeed, a number of industrial worksites, including the Seoul Onsu General Industrial Complex, are located to the southeast of the sampling sites. According to their CPF plot, pollutants from metal-processing facilities and coal fly ash mainly originated in the northwest, likely from emissions from several industrial sites concentrated northwest of the sampling sites. Motor-vehicle-derived pollutants came from most directions, except from the east, in particular the northwest and southwest. The Gyeongin Expressway runs in an east–west direction to the north of the study area, while the First Loop Expressway in the metropolitan region runs in a north–south direction to the west. The two highways also intersect to the northwest.

Pollutants from oil combustion residues arrived mainly from the north. Ojeong General Industrial Complex and many other pollutant-emitting facilities are concentrated to the north of the sampling sites and are considered the source of these pollutants. Soil-derived pollutants are likely to have originated in the northwest. Much construction was underway during the study period, including interior construction, spatial rearrangements, and the construction and demolition of apartment complexes in the northwest. Yellow dust from Incheon and China are assumed to have added to the amount of soil-derived pollutants, as those two locations are northwest of the study area.

Smelter-derived pollutants were observed to originate mainly from the northwest, where industrial sites with metal-processing facilities and those emitting coal fly ash can be found. Pollutants from welding sites appeared to be transported from the southeast, possibly from several worksites that emit air pollutants. The source locations of pollutants from other industries could not be determined as the threshold value (80th percentile) was not met on any of the measurement dates.

From CPF modeling, it was determined that most pollutants came from the northwest, where traffic congestion on the expressways frequently occurs. This releases a high concentration of pollutants that is added to by air pollutants emitted from industrial sites in the northwest and southeast.

## 4. Conclusions

As mentioned in the introduction, air quality studies on PMF models are being conducted mainly in large cities such as Seoul and Jeju Island in the Repubic of Korea. Even in small cities, such as the research areas in this study, the PMF model should be applied to identify the source of pollution.

In this study, PMS-104 samplers were used to collect 74 PM_2.5_ samples at two study sites between 22 April and 3 July 2020, in an effort to identify sources of particulate matter and suggest ways to manage particulate matter concentrations more effectively. A total of 34 chemical components in the collected PM_2.5_ samples were analyzed. PMF modeling showed that secondary aerosols contributed the most to PM_2.5_ pollution (31.09%, 5.73 μg/m^3^), followed by coal-fired boilers (16.9%, 3.11 μg/m^3^), metal-processing facilities and coal fly ash (11.59%, 2.14 μg/m^3^), motor vehicles (10.52%, 1.94 μg/m^3^), oil combustion residues (10.15%, 1.87 μg/m^3^), soil (7.97%, 1.47 μg/m^3^), smelters (6.4%, 1.18 μg/m^3^), welding sites (4.77%, 0.88 μg/m^3^), and other industries (0.7%, 0.11 μg/m^3^).

The PMF model showed an R^2^ value of 87.2. Other papers judged that results are reliable when the R^2^ value is greater than 80, and this study provides results similar to those of other studies. Therefore, the results of this study were deemed reliable. CPF modeling was performed to identify the locations of pollution sources and showed that most pollutants came from areas northwest of the sampling sites. This finding is consistent with the presence of major expressways in the northwest. Furthermore, air-pollutant-emitting worksites are concentrated in the southeast. It was confirmed that most of the coal boiler pollutants were transported from the southeast. This was also consistent with the concentration of air pollutant emission businesses in the southeast.

The emission of pollutants with high contribution rates should be actively regulated and their management prioritized. In particular, the contribution rate was high for “motor vehicle pollutants”, a representative artificial pollutant. This research area is implementing a number of policies to reduce diesel vehicle emissions for automobile pollutants. For example: (1) restriction on the operation of emission grade 5 vehicles from 6 a.m. to 9 p.m. on weekdays; (2) removal of old diesel vehicles from road use; (3) notification of the operation restriction system to owners of vehicles that have not taken measures for low pollution; (4) owners of a vehicle who have not taken measures against low pollution should quickly attach a reduction device. In addition specific mitigation measures are needed, such as no-driving-day schemes, the promotion of electric vehicles, and the installation of smog-reduction devices.

Given the brevity of the study period, the identification of pollutants and the determination of pollutant contributions using PMF modeling were limited to one season. Further research is needed to identify additional sources of pollution and pollutant contribution rates in each season. Moreover, the relatively low number of samples was likely to have affected the reliability of the results; thus, future studies should include more samples and identify more chemical components to enable investigations of seasonal pollution and improve model reliability. The use of potential source contribution function, distributed, and geographic information system models to analyze long-distance pollution sources would also facilitate more effective air quality management and better pollution-related predictions.

The Republic of Korea’s air quality standards, which are laxer than those of other countries, should first be strengthened to manage PM_2.5_ emission sources more effectively. A legal foundation for applying strict particulate matter standards is necessary to ensure public health [24]. The effect of pollutants on air quality can vary according to pollutant concentration and species; thus, measures that specifically address particular risks are needed, such as the Special Act on Fine Dust Reduction and Management and a scheme to control the total pollutant quantity. Currently, the Republic of Korea has few regional measures for addressing air pollution. However, overall air pollution can only be effectively reduced if measures specific for each region are established, with authority granted to the relevant regional management units.

## Figures and Tables

**Table 1:** Air-pollutant-emitting facilities in Bucheon City [8].

Administrative District of Bucheon City	Area (km^2^)	Class 3	Class 4	Class 5	Total
Simgok-dong	2.6		1	4	5
Bucheon-dong	8.1		68	196	264
Sinjung-dong	4.5		3	6	9
Jung-dong	1.8		1	3	4
Sang-dong	3.6				0
Daesan-dong	4.1	1	1	8	10
Sosabon-dong	3.0		3	5	8
Beom-an-dong	5.7			3	3
Seong-gok-dong	7.6		2	3	5
Ojeong-dong	12.4	1	116	330	447
Total	53.4	2	195	558	755

**Table 2:** Summary of PM_2.5_ concentrations and the concentrations of 25 chemical species used for PMF modeling.

Species	S/N Ratio ^(a)^ DL ^(b)^	Concentration (μg/m^3^)				
		Min	25th	50th	75th	Max
PM_2.5_	- -	0.3066	14.5581	17.8298	22.3221	60.7302
Al	7.5 0.0060	0.0030	0.1199	0.2178	0.3749	2.2850
Ti	8.2 0.0004	0.0002	0.0094	0.0139	0.0205	0.0722
Co	7.8 0.3761	0.0000	0.0019	0.0044	0.0102	0.0481
V	7.0 0.0001	0.0001	0.0011	0.0029	0.0047	0.0092
Se	5.5 0.0004	0.0002	0.0009	0.0025	0.0043	0.0110
As	7.5 0.0001	0.0000	0.0017	0.0045	0.0080	0.0284
SiO	2.8 0.0687	0.2172	0.2172	0.2172	2.3837	7.8155
Mg	7.9 0.0075	0.0037	0.0822	0.1579	0.2422	1.0879
Zn	9.0 0.0002	0.0037	0.0440	0.0732	0.0910	0.1295
Br	8.9 0.0002	0.0020	0.0113	0.0186	0.0262	0.0519
Ca	6.7 0.0033	0.0017	0.0156	0.0419	0.0661	0.5559
Pb	8.6 0.0003	0.0001	0.0134	0.0207	0.0304	0.1302
Cr	8.4 0.0004	0.0021	0.0048	0.0061	0.0081	0.0339
Mn	8.1 0.0007	0.0003	0.0106	0.0142	0.0199	0.1024
Fe	8.9 0.0011	0.0102	0.2010	0.2625	0.3296	0.7181
Ni	6.9 0.0001	0.0000	0.0005	0.0012	0.0032	0.0211
Na^+^	5.3 0.0403	0.0448	0.1036	0.1480	0.2819	0.6206
NH_4_^+^	8.3 0.0311	0.0156	0.8914	1.7629	2.9646	8.6075
K^+^	3.6 0.0493	0.0246	0.0246	0.1654	0.2079	0.3672
Cl^−^	2.7 0.0983	0.0492	0.1044	0.1873	0.2932	0.7726
NO_3_^−^	6.4 0.1117	0.0559	0.4143	1.1186	2.5808	12.7241
SO_4_^2−^	3.3 0.1076	0.0538	0.0842	0.2841	0.4689	1.0071
OC	6.9 0.1164	2.1639	6.7140	7.9161	8.9695	13.4190
EC	7.3 0.0196	0.2119	1.1001	1.2565	1.4784	2.4926

^(a)^ S/N ratio: Signal-to-noise ratio, ^(b)^ DL: Detection Limit.

**Table 3:** Contribution of each pollution source on weekdays and weekends (unit: μg/m^3^, %).

Source	Weekdays	Weekends
Secondary aerosols	4.53	7.65
	24.34	42.29
Coal-fired boilers	2.93	3.41
	15.74	18.86
Metal-processing facilities and coal fly ash	1.92	2.49
	10.29	13.74
Motor vehicles	2.36	1.26
	12.66	6.94
Oil combustion residues	2.11	1.49
	11.31	8.22
Soil	2.16	0.36
	11.61	1.97
Smelters	1.33	0.94
	7.13	5.18
Welding sites	1.20	0.36
	6.46	1.97
Other industries	0.09	0.15
	0.47	0.82
Total	18.63100	18.09100

**Table 4:** Contribution of each pollution source by month (unit: μg/m^3^, %).

Source	April	May	June	July
Secondary aerosols	3.61	6.03	4.36	24.35
	22.66	38.14	20.81	62.17
Coal-fired boilers	4.79	2.67	2.39	4.36
	30.04	16.89	11.40	11.13
Metal-processing facilities and coal fly ash	2.00	1.60	2.98	1.86
	12.56	10.13	14.23	4.74
Motor vehicles	0.00	0.48	5.02	3.70
	0.00	3.04	23.98	9.46
Oil combustion residues	0.91	2.01	2.30	2.18
	5.72	12.71	11.00	5.56
Soil	3.52	1.05	0.81	0.00
	22.05	6.64	3.89	0.00
Smelters	0.65	1.43	1.11	1.93
	4.06	9.05	5.29	4.92
Welding sites	0.26	0.40	1.95	0.87
	1.66	2.51	9.33	2.22
Other industries	0.21	0.14	0.01	0.01
	1.33	0.88	0.07	0.02
Total	15.95 100	15.82 100	20.94 100	39.16 100

## Data Availability

Not applicable.
